# Interactions With Histone H3 & Tools to Study Them

**DOI:** 10.3389/fcell.2020.00701

**Published:** 2020-07-31

**Authors:** William A. Scott, Eric I. Campos

**Affiliations:** ^1^Genetics & Genome Biology Program, The Hospital for Sick Children, Toronto, ON, Canada; ^2^Department of Molecular Genetics, University of Toronto, Toronto, ON, Canada

**Keywords:** H3.1, H3.3, histone, nucleosome, chromatin, epigenetic, proteomic

## Abstract

Histones are an integral part of chromatin and thereby influence its structure, dynamics, and functions. The effects of histone variants, posttranslational modifications, and binding proteins is therefore of great interest. From the moment that they are deposited on chromatin, nucleosomal histones undergo dynamic changes in function of the cell cycle, and as DNA is transcribed and replicated. In the process, histones are not only modified and bound by various proteins, but also shuffled, evicted, or replaced. Technologies and tools to study such dynamic events continue to evolve and better our understanding of chromatin and of histone proteins proper. Here, we provide an overview of H3.1 and H3.3 histone dynamics throughout the cell cycle, while highlighting some of the tools used to study their protein–protein interactions. We specifically discuss how histones are chaperoned, modified, and bound by various proteins at different stages of the cell cycle. Established and select emerging technologies that furthered (or have a high potential of furthering) our understanding of the dynamic histone–protein interactions are emphasized. This includes experimental tools to investigate spatiotemporal changes on chromatin, the role of histone chaperones, histone posttranslational modifications, and histone-binding effector proteins.

## Introduction

Chromatin is composed of DNA and associated proteins, of which histones are prominent. Histones and DNA assemble to form repetitive units known as nucleosomes. Each nucleosome organizes a stretch of ∼147 bp of DNA wrapped around a histone octamer (Luger et al., 1997). The octamer is, in turn, composed of a central (H3-H4)_2_ tetramer, flanked by two H2A-H2B dimers. Nucleosomal arrays give rise to an 11 nm fiber that resembles “beads on a string,” as seen in early micrographs of chromatin (Olins et al., 1976). Histone proteins are heavily modified through combinatorial posttranslational modifications (PTMs), especially over their N-terminal tails that protrude from the nucleosomal core (Huang et al., 2015a; Andrews et al., 2016). These modifications influence local protein–protein interactions (PPIs) and chromatin structures, and consequently have important implications on DNA accessibility, transcription, repair, and replication. A large number of histone PTMs thereby correlate, or anti-correlate, with various biological outputs (Campos and Reinberg, 2009; Allis and Jenuwein, 2016).

This review highlights current models of H3.1 and H3.3 dynamics, namely: the histones, their PTMs, deposition pathways, and cell cycle dynamics. Each section provides an overview of the techniques used to formulate the models.

## Histone Variants and Post-Translational Modifications

Certain histone variants are believed to influence the biophysical characteristics of nucleosomes (Campos and Reinberg, 2009), thereby relaying functional consequences on chromatin. Histone H2A has a relatively high number of variants, while histones H2B and H4 have undergone little evolutionary divergence–likely reflecting their positions within the nucleosome and their roles in stabilization of the nucleosome core particle (Henikoff and Smith, 2015). There are, however, a large number of histone H3 variants in humans, namely H3.1, H3.2, H3.3, H3t/H3.4, H3.5, H3.Y, H3.X, CENP-A, the more recently proposed H3.3-like H3.6 and H3.8, as well as the H3.1-like H3.7 (Franklin and Zweidler, 1977; Earnshaw and Rothfield, 1985; Albig et al., 1996; Wiedemann et al., 2010; Schenk et al., 2011; Taguchi et al., 2017). Of these, the replication-coupled H3.1, and replication-independent H3.3 variants are arguably some of the better-studied histone proteins and hence the focus herein. A broader overview of known histone variants is available elsewhere (Talbert et al., 2012; Biterge and Schneider, 2014; Henikoff and Smith, 2015).

**FIGURE 1:**
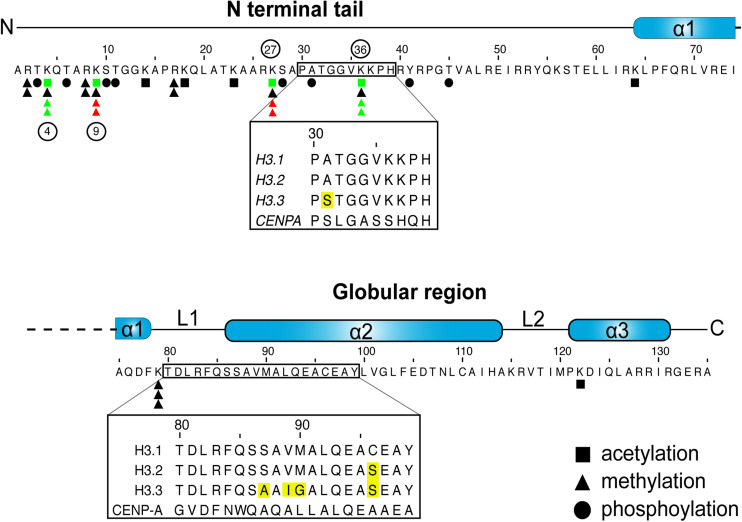
H3 sequence and posttranslational modifications. Graphic representation of human H3.1 primary sequence and secondary structures. Inset boxes denote areas that differ between the H3.1 and H3.3 variants, with sequence differences highlighted in yellow. Residues that are subject to acetylation, methylation or phosphorylation are denoted as such (Paulson and Taylor, 1982; Bernstein et al., 2005; Wang et al., 2008; Zhao and Garcia, 2015). The number of triangles marks the maximum number of methyl marks that can be installed. Select key marks that generally correlate with transcriptional repression (red) and activation (green), and that are discussed in the review, are also noted (Rea et al., 2000; Bernstein et al., 2005; Hake et al., 2006; Wang et al., 2008; Margueron et al., 2009). The phosphorylation of residue 31 is exclusive to H3.3 (Hake et al., 2005).

H3.1 and H3.2 differ by a single amino acid at residue 96 (Figure 1; Hake and Allis, 2006). Both are expressed in S-phase (Wu et al., 1982; Mendiratta et al., 2019) and deposited on replicating DNA (Tagami et al., 2004). They are, therefore, considered to be replication-coupled (RC) histones. Reflecting the need for considerable histone production during DNA replication, RC histones are expressed from histone gene clusters in S-phase (Wu and Bonner, 1981). As such, H3.1 predominates in cycling cells (Wu et al., 1982; Marzluff et al., 2002).

Conversely, the H3.3 variant (encoded by *H3F3A* and *H3F3B*) is expressed at low levels throughout interphase (Wu and Bonner, 1981; Mendiratta et al., 2019) to maintain proper nucleosome density as histones turnover. It is thus referred to as replication-independent (RI). H3.3 is particularly enriched over actively transcribed genes, but is also deposited over repetitive DNA elements, such as pericentromeric regions and telomeres (Ahmad and Henikoff, 2002; Drane et al., 2010; Goldberg et al., 2010). It accumulates in terminally differentiated cells (Grove and Zweidler, 1984), and is also the only non-centromeric H3 variant in some species (e.g., yeast within the Ascomycota phylum) (Postberg et al., 2010; Talbert and Henikoff, 2010). Astonishingly, the H3.3 RI histone differs from H3.1 by only five residues (Hake and Allis, 2006). Yet, these minute differences are sufficient to confer specificity to distinct interacting proteins, such as histone chaperones (Elsasser et al., 2012; Ricketts et al., 2015).

Histone pools are exquisitely regulated at the transcriptional and posttranscriptional levels. Reduced histone transcription disturbs the cell cycle (Nelson et al., 2002) and excessive production of histones outside of S-phase leads to chromosomal instability (Gunjan and Verreault, 2003), partly through a stoichiometric imbalance (Meeks-Wagner and Hartwell, 1986). Soluble histones pools are also kept in check by certain histone chaperones (Groth et al., 2005; Cook et al., 2011). Histones are then deposited onto DNA to form nucleosomes and specialize local chromatin regions (Campos and Reinberg, 2009). Once on chromatin, histones remain highly dynamic, even when deposited in heterochromatic regions (Consortium et al., 2007; Deal et al., 2010). Such dynamics are particularly evident as DNA is transcribed, repaired, replicated, and condensed.

A large number of histone residues are subject to various PTMs, including methylation, acetylation, and phosphorylation, to name but a few [see Zhao and Garcia (2015) for a comprehensive list]. Combinatorial histone PTMs, particularly over the lysine-rich N-terminal histone tails influence local chromatin structures and dynamics, and often correlate with transcriptional status (Zhao and Garcia, 2015; Allis and Jenuwein, 2016; Figure 1).

Histone acetylation has long been shown to correlate with active gene transcription (Allfrey et al., 1964; Gorovsky et al., 1973; Davie and Candido, 1978; Chahal et al., 1980). The PTM neutralizes the positive charge on the ε-amino group of lysine residues, leading to numerous downstream events. For one, lysine acetylation on histone proteins is believed to counteract chromatin compaction (Wong and Marushige, 1976; Wallace et al., 1977; Nelson et al., 1978; Simpson, 1978; Vidali et al., 1978; Annunziato et al., 1988; Tse et al., 1998). For example, the acetylation of lysine 122 on H3 (H3K122ac) has been found to destabilize nucleosomes by disrupting histone–DNA interactions (Tropberger et al., 2013). Beyond direct biophysical effects, the binding of numerous effector proteins that “read” modified histones further influences chromatin structures. For example, acetyl marks are recognized by the bromodomain, YEATS, or plant homeodomain (PHD) of some chromatin-associated proteins. Similarly, methylated lysines are recognized by a “Royal Family” (tudor, MBT, chromodomain, PWWP), as well as numerous other domains, including PHD, WD40, ankyrin repeats, BAH, and ADD (see Patel and Wang, 2013; Andrews et al., 2016).

“Reader proteins” exert further effects on chromatin. The H3K9me2/3 and H3K27me2/3 marks, for example, correlate with chromatin compaction and transcriptional repression as a result of some proteins that bind these marks. H3K9me3 enriches at constitutive heterochromatic regions, such as pericentric chromatin and telomeres (Talbert and Henikoff, 2006). The mark is catalyzed by the SUV39H1/2 histone methyltransferases in humans (Rea et al., 2000), which is, in turn, recognized by the heterochromatin protein 1 (HP1) via its chromodomain (Smothers and Henikoff, 2000; Lachner et al., 2001). This process is sustained through a positive feedback loop, where HP1 re-recruits SUV39H1/2 to propagate the mark (Talbert and Henikoff, 2006). The HP1 protein further phase separates—that is, adopts liquid-like properties to form a membraneless compartment—thereby driving chromatin compaction (Larson et al., 2017; Strom et al., 2017).

Similarly, the H3K27me3 mark—which is particularly enriched over facultative heterochromatin—also spreads through a positive feedback loop driven by the polycomb repressive complex 2 (PRC2). The EED subunit of this complex binds the H3K27me3 mark to allosterically activate the EZH2 catalytic subunit, thereby propagating the mark to neighboring nucleosomes (Margueron et al., 2009; Oksuz et al., 2018). Our current understanding of polycomb proteins is evolving at a fast pace, and is well discussed in recent publications (Cheutin and Cavalli, 2019; Laugesen et al., 2019; van Mierlo et al., 2019; Yu et al., 2019; Chammas et al., 2020).

Not all histones marks are believed to alter chromatin structures but can still influence biological events by preventing or promoting interactions with other proteins. For example, H3K4me2/3 enriches near the transcriptional start site (TSS) of actively transcribed genes (Bernstein et al., 2005; Kim et al., 2005; Roh et al., 2006). The mark alone fails to stimulate transcription *in vitro* (Pavri et al., 2006), but does prevent the installment of repressive H3K9 and H3K27 methyl marks on the same histone tail (Binda et al., 2010; Schmitges et al., 2011; Voigt et al., 2012). Just as importantly, the mark facilitates a number of events, including transcriptional initiation, splicing, and even termination (Sims et al., 2007; Vermeulen et al., 2007; Terzi et al., 2011).

H3K36me2/3 is another important mark that correlates with gene expression, but enriches over transcribed gene bodies (Bannister et al., 2005). Like H3K4me3, it is also inhibitory toward PRC2 activity on the same histone tail (Schmitges et al., 2011; Yuan et al., 2011; Voigt et al., 2012). The mark has been notably associated with transcriptional elongation, splicing, and the inhibition of cryptic transcription (Carrozza et al., 2005; Keogh et al., 2005; Luco et al., 2010).

Beyond the examples listed above, innumerable combinatorial PTMs coupled to dynamic effects of histone “writers,” “erasers,” and “readers” add further complexity. Therefore, a single histone mark may be impactful in numerous ways and the study of such plasticity requires suitable technologies. New sequencing-based techniques have had an immense impact on that front (Dirks et al., 2016; Nakato and Sakata, 2020; Stewart-Morgan et al., 2020), as have recent developments in mass spectrometry (MS)-based pipelines (Gingras et al., 2007; Eubanks et al., 2017; Simithy et al., 2018; Sequeira and Vermeulen, 2019; Samavarchi-Tehrani et al., 2020). There is, however, also great excitement on ever evolving molecular and biochemical techniques to study histone dynamics, their marks, and PPIs.

## Tools to Study Interactions With Histone PTMs

**FIGURE 2:**
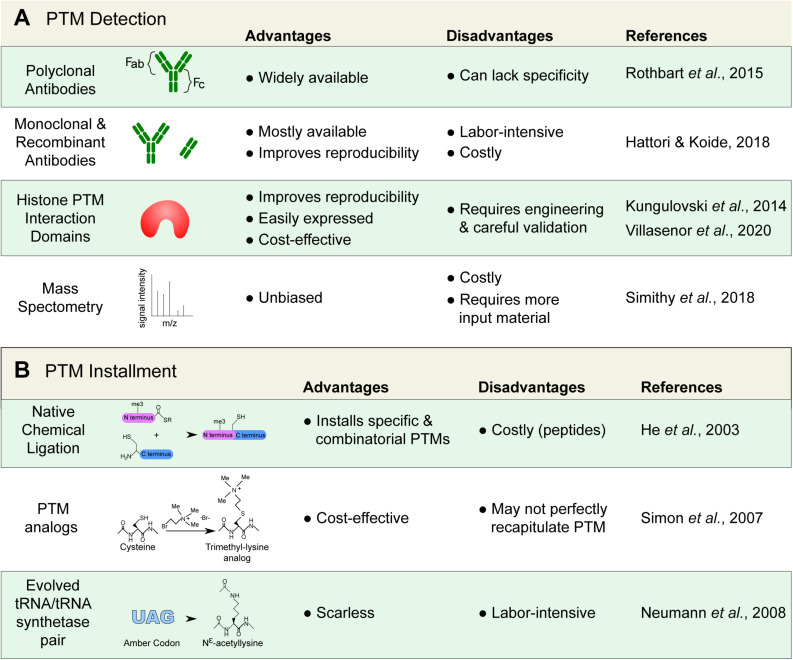
Detecting and installing histone posttranslational modifications (PTMs). **(A)** Common tools used to detect histone PTMs. Antibodies remain standard; however, they can lack specificity and require proper validation. Alternatives, such as recombinant antibodies and histone PTM interaction domains are increasingly available. Mass spectrometry can also provide an unbiased detection. **(B)** Techniques used to install and study specific histone marks. Modified histones are important for the study of histone protein–protein interactions that are modulated by PTMs.

**FIGURE 3:**
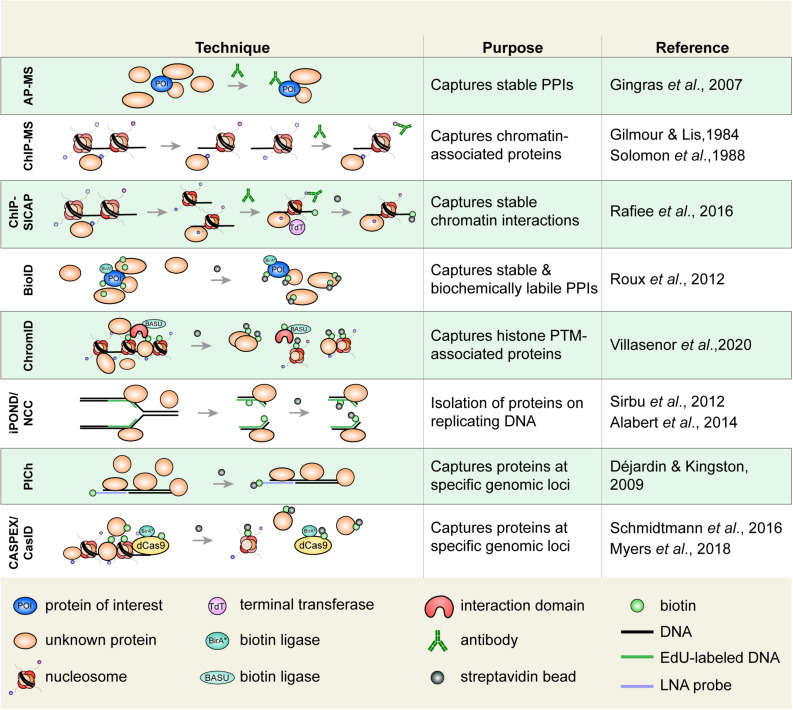
Select techniques used to study histone or chromatin-associated proteins. Affinity purification-mass spectrometry (AP-MS) is used to isolate biochemically stable protein–protein interactions. Chromatin immunoprecipitation (ChIP), in which an epitope of interest is isolated from sheared chromatin fragments, can be coupled to MS to identify associated proteins. ChIP-SICAP uses an additional DNA biotinylation step to wash proteins not directly bound to chromatin. Proximity-dependent labeling techniques are increasingly used to capture stable and biochemically labile protein interactions. Biotin identification (BioID) uses a biotin ligase fused to a protein of interest to biotinylate proximal proteins. Biotinylated proteins are captured on streptavidin beads. ChromID is similar to BioID in that a biotin ligase, BASU, is fused to a histone-binding domain to biotinylate proteins near a PTM of interest. Isolation of proteins on nascent DNA (iPOND)/nascent chromatin capture (NCC), are used to isolate proteins associated with replicating DNA. Cells are pulsed with a thymidine analog (e.g., EdU), which is incorporated on replicated DNA enabling the isolation of replicated chromatin fragments. Proteomics of isolated chromatin (PICh) is used to identify proteins that are bound to a specific genomic region. A biotin-tagged locked nucleic acid (LNA) probe is used to isolate chromatin fragments with DNA complementary to the probe. In CASPEX/CasID, catalytically dead Cas9 (dCas9) is fused to APEX or BirA*, respectively, to perform biotin labeling of a specific genomic locus. PPIs, protein–protein interactions.

Numerous techniques that are used to study histone PTMs rely on antibodies to recognize (Figure 2A) or isolate associated proteins or to map their genomic location (Figure 3). Alternatives, such as recombinant antibodies and purified histone modification interaction domains (HMIDs), are being developed and show promise (Kungulovski et al., 2014; Hattori and Koide, 2018). For example, a recently engineered HP1 chromodomain is reported to surpass antibodies in avidity when binding H3K9me3, without losing specificity (Albanese et al., 2020). Once validated, HMIDs can be expressed and purified at the required scale, eliminating lot variations associated with polyclonal antibodies. Until these alternatives become commonplace, antibody use remains the standard. It is thereby critical to emphasize the need to thoroughly validate the specificity of antibody or binding module (Rothbart et al., 2015).

Of the various antibody-based techniques used to study histone PTMs, chromatin immunoprecipitation (ChIP), has been particularly powerful (Gilmour and Lis, 1984; Hebbes et al., 1988; Solomon et al., 1988). Coupled to microarray (ChIP-chip), sequencing (e.g., ChIP-SAGE), and later next-generation sequencing (ChIP-seq), ChIP-based techniques facilitated the genomic mapping of histone PTMs, and studies that correlated histone PTMs with various chromatin states or biological effects (Robyr et al., 2002; Roh et al., 2004; Barski et al., 2007). In addition to mapping histones, PTMs, and histone binding-proteins, an increasing number of variations on the ChIP technique are used to investigate histone dynamics [e.g., ChOR-seq, SCAR-seq – see Stewart-Morgan et al. (2020)]. Furthermore, direct analysis of ChIP material (e.g., ChIP-western or ChIP–MS) is possible, and can inform on protein associations within chromatin fragments containing specific histone PTMs (Ji et al., 2015). As with all ChIP-based experiments, the technique is limited by antibody specificity, the abundance of the epitope, and downstream detection (e.g., MS). It, however, is a relatively accessible technique that is applicable toward different ends.

Histone peptides or nucleosomal particles containing specific PTM(s) are perhaps more commonly immobilized to isolate and identify histone readers. There are different approaches that are used to install PTMs on histone proteins *in vitro* (Figure 2B). Histones can, of course, be enzymatically modified *in vitro*, but that risks yielding a mixture of modified and unmodified histones, modifying more than one residue, or results in multiple states (e.g., mono-, di-, tri- lysine methylation). Small histone peptides containing specific marks can also be chemically synthesized, but they then lack the important nucleosomal context.

It is possible to obtain homogenously modified histones by chemically ligating synthetic histone tails (containing specific marks) to tailless recombinant histones (He et al., 2003; Shogren-Knaak et al., 2003). In this system, a synthesized C-terminal histone globular region containing an N-terminal cysteine and a synthesized N-terminal histone tail containing a C-terminal thioester are spontaneously ligated to produce full-length protein. The technique has helped elucidate mechanisms of chromatin readers, such as that of the BPTF protein, which simultaneously binds the H3K4me2/3 and H4K16ac marks (Ruthenburg et al., 2011). There are also strategies to install site-specific PTM analogs. In this approach, the residue of interest is mutated to a cysteine and an aminoethylation reaction ligates a PTM analog. This semisynthetic method has generated numerous PTM mimics, including methyl lysine analogs (often referred to as MLAs) and acetyl lysine mimics (Simon et al., 2007; Huang et al., 2010). Though the PTM analogs allow for a qualitative analysis of binding proteins, the sulfide substitution on the side chain has been suggested to affect binding strength of certain interactions (Seeliger et al., 2012; Chen Z. et al., 2018). Nevertheless, the semisynthetic strategies remain a formidable tool (reviewed by Holt and Muir, 2015).

Biorthogonal systems allow for the expression of “scarless” recombinant histones. They have been developed to express dedicated tRNA/tRNA-synthetase pairs to expand the genetic code and incorporate modified amino acids into recombinant proteins expressed in bacteria (Neumann et al., 2008), or even exogenous histones expressed in mammalian cells (Elsasser et al., 2016). While an effective system, the evolution of tRNA/tRNA-synthetase pairs is labor-intensive. Regardless of the process used, the modified histones can be assembled into nucleosomes, immobilized to capture interacting proteins, or used to study histone PTM-protein interactions.

Candidate proteins can also be tested against multiple histone PTMs to survey the marks they recognize. Peptide arrays contain short, synthetic histone tails etched on a solid surface. By containing different PTMs, each polypeptide allows researchers to discern the ability of a reader to bind to specific marks or combinations thereof (Mauser and Jeltsch, 2019). However, because these arrays only contain a portion of the histone tail, they may skew binding. As with all screening techniques, a careful validation is required. The technique, however, is often used and has generated insightful data pertaining to histone PTM-binding proteins. A refined quantitative approach using immobilized peptides, combined with stable isotope labeling by amino acids in cell culture (SILAC) was used to distinguish specific binders over background binders (Vermeulen et al., 2007, 2010). By incubating modified peptide with lysates from cells grown in the presence of heavy isotopes, and unmodified peptides with lysates from cells grown in the presence of light isotopes, the general transcription factor TFIID was found to associate with the H3K4me3 mark using mass spectrometry (Vermeulen et al., 2007).

Newer technologies also allow for the identification of histone PTM binding proteins. Proximity-dependent labeling approaches (Martell et al., 2012; Roux et al., 2012)—discussed in greater detail below—have been revolutionizing the proteomics field. A clever take on the technique, called ChromID uses engineered protein modules to bind histone PTMs (Villasenor et al., 2020). Further coupled to a promiscuous biotin ligase enzyme, the module biotinylates proteins that are directly or indirectly associated with the associated histone PTM. The biotinylated proteins are then captured on streptavidin beads under denaturing conditions, and identified by MS. To further capitalize on the technique, a larger collection of interacting modules will now need to be developed.

While several histone marks are relatively well studied, the majority arguably remain poorly characterized. Screening tools like ChromID will surely prove immensely beneficial in dissecting the roles of histone PTMs, and perhaps even combinations thereof.

Once the interacting proteins have been identified, a battery of biophysical tools are used to validate the interaction and characterize its thermodynamic properties. This establishes binding affinities and can provide additional information, such as protein stoichiometry within a complex. Common techniques include isothermal titration calorimetry (ITC), dynamic light scattering, analytical ultracentrifugation (AUC), bio-layer interferometry (BLI), and microscale thermophoresis (MTS), to name a few (see Ausio, 2000; Lewis and Murphy, 2005; Concepcion et al., 2009; Stetefeld et al., 2016; Asmari et al., 2018).

It is important to emphasize that histone PTMs are found in thousands of combinations throughout the genome. To add further complexity, these marks are constantly added, removed, and bound by other proteins. Antibody-based techniques are instrumental to their study, but an increasing number of innovative technologies also continue to facilitate research through such plasticity.

## Histone Protein–Protein Interactions: From Protein Translation to Nuclear Import

Guided by biochemical and cellular approaches, two different models (which may not necessarily be mutually exclusive) have been proposed to explain the pathway by which newly synthesized histones are folded, processed, and imported into the nucleus [discussed in Grover et al. (2018) and Pardal et al. (2019)]. In both models, new histones are folded by molecular chaperones and imported into the nucleus while bound by importin-4. The two models mainly differ in the order of events, and their subcellular localization.

In the first model, affinity purification of epitope tagged cytoplasmic H3 followed by column chromatography resolved a number of core histone protein complexes that hint toward an organized processing and nuclear import of newly synthesized histones (Campos et al., 2010; Alvarez et al., 2011). Newly translated H3 and H4 monomeric units are first folded by molecular chaperones (complex Ia: H3-HSC70; complex Ib: H4-HSP90-HSC70) before their assembly into H3-H4 dimers by HSP90 and tNASP (complex II). Complex III involves processing by sNASP and the HAT1 holoenzyme. NASP proteins help fold the H3-H4 histones (Bowman et al., 2017), facilitate H4 acetylation by HAT1 (Campos et al., 2010), and regulate soluble H3-H4 levels (Cook et al., 2011). Once processed, the H3-H4 dimers are transferred to the ASF1 histone chaperone for import into the nucleus in complex IV (H3-H4-ASF1-importin 4). ASF1 then transfers the histones to other histone chaperones that deposit them onto DNA.

There are a number of important intricacies regarding protein isoforms and the sequential addition of histone PTMs (Alvarez et al., 2011). Some were captured by alternative means since protein chromatography resolves biochemically stable protein complexes. For example, the isolation and proteomic analysis of polysomes containing histone polypeptides undergoing translation allowed for the identification of SETDB1 (KMT1E/ESET) as the enzyme responsible for the monomethylation of lysine 9 on a subset of new H3 proteins (Rivera et al., 2015). This nuance is important since the mark is believed to prime replicating chromatin for heterochromatin formation (Loyola et al., 2006).

The second histone nuclear import model is based on cellular technique known as rapamycin-activated protease through induced dimerization and release of tethered cargo (RAPID-release) (Apta-Smith et al., 2018). In this technique, new histones are tethered to the cytosolic side of the outer mitochondrial membrane, released through rapamycin-activated cleavage of the tethering moiety, and tracked using a fluorescent tag. While the effect of tethering histones is unclear, the study clearly illustrates flexibility in the histone processing pathway. The experimental pipeline shows that H3 and H4 monomers can be imported into the nucleus while directly associated with importin-4. H3-H4 dimer assembly and histone chaperoning then occurs in the nucleus. The two models are not necessarily mutually exclusive, but the reports emphasize the need to further explore the pre-deposition histone pathways and highlights the benefits of considering multiple experimental approaches.

## Histone Deposition on Chromatin: Vive la Diversité!

Two of the three main H3-H4 histone deposition pathways were elucidated long before the pre-deposition processing pathways above. Biochemical approaches and *in vitro* systems were critical toward the identification of variant-specific histone chaperones that deposit histones on DNA. ASF1-bound H3-H4 histones can be transferred to the CAF-1, HIRA, and DAXX histone chaperones for deposition on DNA.

Chromatography-based fractionation of HEK293 nuclear extracts enabled the isolation of histone deposition activity that occurred on replicating DNA. Histone deposition was tested from fractions, using an *in vitro* replication system in the presence of soluble histones. This led to the identification of the replication-coupled Chromatin Assembly Factor 1 (CAF-1) (Smith and Stillman, 1989). CAF-1 is a three-subunit H3-H4 histone chaperone. It is coupled to DNA replication because of its interaction with the proliferating cell nuclear antigen (PCNA) processivity ring, via a PCNA interacting peptide (PIP) motif (Rolef Ben-Shahar et al., 2009). ASF1-bound H3-H4 dimers are first transferred to CAF-1 through direct interactions between ASF1 and the p60 subunit of CAF-1 (Tyler et al., 2001; Mello et al., 2002). Histone deposition is then a matter of thermodynamics (Das et al., 2010; Liu et al., 2012; Sauer et al., 2018).

Gel filtration, relative amino acid composition, and AUC analyses showed that a single ASF1 binds an H3-H4 dimer, occluding histone tetramerization prior to deposition (English et al., 2005), something that was further confirmed by X-ray crystallography (English et al., 2006; Natsume et al., 2007). The CAF-1 winged helix domain binds DNA and promotes the tetramerization of two H3-H4 dimers while forming nucleosomes by depositing the (H3-H4)_2_ tetramer on DNA (Liu et al., 2012; Mattiroli et al., 2017; Sauer et al., 2018). This stepwise transfer of H3-H4 from ASF1 to CAF-1 and, ultimately, DNA is explained by a “nucleosome assembly funnel” (Das et al., 2010). In this model, free histones have high free energy and are handed from chaperone to chaperone to be assembled into stable intermediates until ultimately being transferred to DNA, the state with the lowest free energy. The dissociation constant of histones bound to either ASF1 or CAF-1 is in the low nanomolar range (Donham et al., 2011; Liu et al., 2012). However, fluorescent anisotropy and electrophoretic mobility shift assays (EMSAs) determined that yeast Asf1 and CAF-1 preferentially associate when Asf1 is pre-bound to H3-H4 (Liu et al., 2012). Förster resonance energy transfer (FRET) experiments, in which equimolar amounts of donor- and acceptor-labeled H4 were mixed to measure tetramer formation, resulted in nearly identical fluorescent emission spectra for (H3-H4)_2_ bound to CAF-1 or DNA, suggesting that CAF-1 primes H3-H4 for deposition on DNA (Liu et al., 2012; Sauer et al., 2018). The resulting tetrasome folds some 80 bp of DNA until H2A-H2B dimers complete the nucleosome (Brower-Toland et al., 2002; Sauer et al., 2018).

Unlike CAF-1, the HIRA histone chaperone easily deposits histones on static DNA templates *in vitro* (Ray-Gallet et al., 2002). Affinity purification of epitope-tagged H3.1 and H3.3 from mammalian cells found the CAF-1 and HIRA protein complexes to act as their respective histone chaperones (Tagami et al., 2004). In mammals, it is the ubinuclein-1/2 subunit that confers specificity toward H3.3, as shown via biophysical and structural analyses (Ricketts et al., 2015). HIRA notably operates over transcribed genes, where nucleosomal histones are disrupted (Goldberg et al., 2010; Sarai et al., 2013).

Biochemical fractionation of affinity purified H3.3-associated proteins also led to the identification of yet another H3.3 histone chaperone, DAXX, and its binding partner ATRX (Drane et al., 2010; Lewis et al., 2010). Protein crystallization found the H3.3-specific G90 residue to be key for the interaction with DAXX (Elsasser et al., 2012). Unlike HIRA, DAXX-ATRX mainly deposits H3.3 over repressed, repetitive DNA elements, where nucleosomes are important for stability.

**FIGURE 4:**
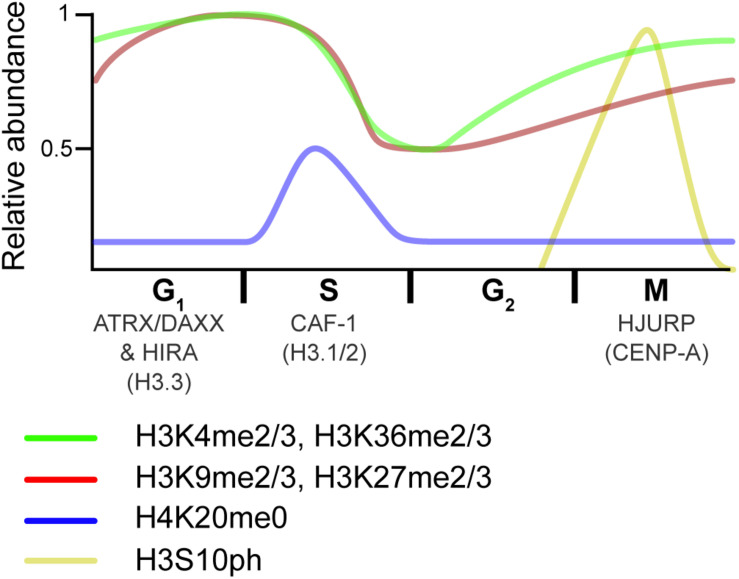
Overview of histone dynamics throughout the cell cycle. Replication-coupled H3.1 and H3.2 are deposited by CAF-1 during DNA replication. New histones transiently lack PTMs on H4K20 (blue). Meanwhile, preexisting histone marks become diluted, as old histones are recycled on the two new DNA strands. As the cell progress into G_2_, chromatin matures, and marks associated with active transcription (green) are reset via the transcriptional process. In contrast, repressive marks (red) begin to epigenetically spread from old to new histones. As the cell transitions from G_2_ to M, histones are transiently phosphorylated (yellow), a mark that is thought to recruit proteins that aid in condensation. HJURP deposits the H3 centromeric variant CENP-A at the end of the cell cycle/beginning of the next one. The RI H3.3 variant is deposited at transcribed genes by the HIRA histone chaperone complex and over repetitive DNA regions by the ATRX–DAXX complex in interphase. Relative PTM abundance is denoted by the height of the curve and based on data from references provided in the main text. For simplicity, new histones are indicated by the H4K20me0 mark, but also contain H3K9me1 and H4K5/K12ac.

Biochemical fractionations also helped identify the histone chaperone for the centromeric H3 variant, CENP-A: HJURP; while IF-based studies showed that CENP-A deposition occurs in late M/early G_1_ (Jansen et al., 2007; Dunleavy et al., 2009). As such, CAF-1, HIRA, DAXX, and HJURP deposit different H3 variants, at specific times, and over different genomic regions (Figure 4). While these deposition pathways are well established, new ones are seeing light (see section “Histone Recycling and Tools to Study Interactions at Replication Forks”).

## Techniques to Study Histone Occupancy and Deposition

To test for histone chaperone activity, proteins must be shown to specifically bind histones, promote histone deposition on DNA without the use of ATP, and dissociate from the final product (*i.e.*, nucleosome) (Gurard-Levin et al., 2014; Hammond et al., 2017; Grover et al., 2018; Ricketts et al., 2019). *In vitro* and *in vivo* techniques are available for this purpose.

*In vitro* methods used to assess histone deposition on naked DNA are well established (Germond et al., 1975; Noll et al., 1975; Loyola et al., 2004). These minimally contain free histones, a histone chaperone, and a DNA template, which are incubated for a fixed period of time near physiological salt concentrations. If using a short piece of linear DNA (i.e., accommodates a single nucleosome), the assembly can be observed by techniques as simple as EMSA (Laskey et al., 1978). If using longer DNA templates (e.g., a plasmid), other techniques are better suited. Micrococcal nuclease (MNase) preferentially cleaves internucleosomal DNA, and a limited digest releases mononucleosomes and multimers thereof (Noll, 1974). Deproteinated DNA can then be separated by gel electrophoresis for assessment. MNase digests are particularly informative on the quality of nucleosome assembly and nucleosome spacing (Lusser and Kadonaga, 2004), but other techniques better assess the proportion of nucleosome assembly. The DNA supercoiling assay, in turn, measures topological changes due to the formation of nucleosomes on a closed circular DNA template. This assay directly quantifies the extent of nucleosome assembly because each nucleosome adds a superhelical turn (Germond et al., 1975). The assay demonstrated the histone chaperone activity of the first histone chaperone to be isolated from extracts (Laskey et al., 1978), and remains a gold standard in the field.

It is also possible to assess histone occupancy *in vivo*, notably by ChIP. At a more global level, MNase digestion can be performed on genomic chromatin from intact nuclei. It is even possible to probe specific genomic regions to assess their relative accessibility (Wu et al., 1979). A newer technique is, however, more commonly used to assess chromatin accessibility at the genome-wide level. The assay for transposase-accessible chromatin coupled to sequencing (ATAC-seq), is a method that involves the fragmentation and tagging (tagmentation) of the genome with sequencing adaptors using the Tn5 transposase. Sequencing reads then reveal genomic regions that are highly represented and, thus, within more accessible chromatin (Buenrostro et al., 2013).

There are, of course, several other specialized techniques to quantify and qualify nucleosome assembly, such as electron microscopy (EM) and single-molecule techniques (see Duzdevich and Greene, 2013; Schwartzman and Tanay, 2015; Senapati et al., 2015).

## Histone Recycling and Tools to Study Interactions at Replication Forks

**FIGURE 5:**
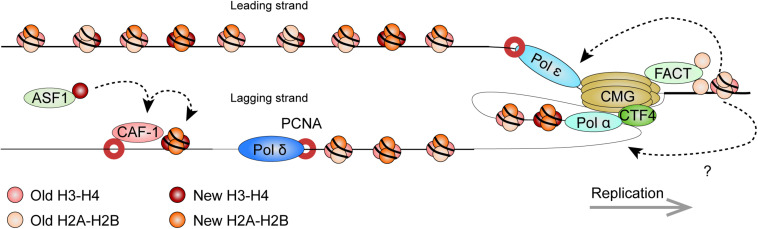
Model: Histone dynamics at the replication fork. The replication machinery encounters nucleosomes as it progresses. As this occurs, the MCM2 subunit of the CDC45/MCM2-7/GINS (CMG) DNA helicase cooperates with FACT to evict nucleosomal histones. The evicted histones, containing a panoply of PTMs segregate in nearly equal amounts for deposition onto both new DNA strands. Histone redeposition involves histone chaperone activity of polymerases behind the fork. CAF-1 further associates with PCNA to deposit newly synthesized histones alongside the recycled ones. It receives new histones from ASF1. Circles depict histone pairs, for simplicity. References are provided in the main text.

While the deposition of new histones via CAF-1 is well-established, exciting findings are beginning to shed light on the eviction, segregation, and redeposition of pre-existing nucleosomal histones [see Grover et al. (2018); Sauer et al. (2018), and Stewart-Morgan et al. (2020) for detailed updates on the topic]. When DNA is copied, preexisting nucleosomal histones dissociate from the replicating DNA strand (eviction) to redistribute on both nascent DNA strands (segregation) and form new nucleosomes (redeposition) (Figure 5). This histone recycling process is extremely complex and requires precise steps to ensure that epigenetic information is maintained while faithfully replicating DNA. Studying histone recycling at replication forks is challenging because of the dynamic nature of the process. There is also a need to uncouple the deposition of recycled, pre-existing nucleosomal histones from that of their newly synthesized counterparts. While biochemical approaches, such as those discussed above, continue to better our understanding of histone recycling, so are new emerging technologies.

The eukaryotic replisome is spearheaded by the CDC45/MCM2-7/GINS (CMG) DNA helicase that unwinds the DNA double helix (Gambus et al., 2006; Moyer et al., 2006). As the helicase tracks along chromatin, it also makes contact with nucleosomes ahead of the replication machinery and plays an important role in histone eviction. The minichromosome maintenance 2 (MCM2) subunit harbors a conserved N-terminal region that imparts histone chaperone activity (Ishimi et al., 2001; Foltman et al., 2013; Huang et al., 2015b). The eviction process requires the facilitates chromatin transcription (FACT) histone chaperone. Pull-down experiments in yeast showed that FACT and Mcm2 cooperatively bind histones (Foltman et al., 2013). Strains with mutations affecting Mcm2 histone-binding residues suffered from a loss of heterochromatin, highlighting its importance in histone recycling. Crystallography and single molecule assays using optical tweezers show that the SUPT16H subunit of FACT (Spt16 in yeast) can displace and tether nucleosomal H2A-H2B dimers while stabilizing the (H3-H4)_2_ tetramer (Chen P. et al., 2018; Wang et al., 2018; Liu et al., 2020). Further structural work showed that MCM2 in turn associates with (H3-H4)_2_ surfaces that normally interact with DNA, thereby shielding the tetramer from aberrant interactions (Huang et al., 2015b). Altogether, this suggests a synergistic role for MCM2 and FACT in the disassembly of nucleosomes on replicating DNA.

By labeling newly replicated DNA, and separating replicated and non-replicated chromatin on density gradients, early studies showed that pre-existing nucleosomal histones segregate to both leading and lagging strands (Jackson et al., 1975). Elegant EM visualization of replicating minichromosomes further found that nucleosomes rapidly reform on nascent chromatin near the replication fork (Sogo et al., 1986). More recently, SILAC, coupled to a controlled pulse-chasing of pre-existing or new histones, further demonstrated that histones are predominantly recycled as (H3-H4)_2_ tetrameric and H2A-H2B dimeric units (Xu et al., 2010). Curiously, H3.3-containing tetramers were more apt to dissociate into dimers thereby allowing intermixing with new histones. Meanwhile, H2A-H2B dimers readily re-associated with new and old (H3-H4)_2_ tetramers.

There is now exciting new data regarding the mechanisms by which nucleosomal histones are evicted, segregate, and reassemble on the newly replicated DNA. Sister chromatids after replication by DNA sequencing (SCAR-seq) in mouse ESCs (mESCs) demonstrated parental histones segregated with a slight preference for the leading strand (Petryk et al., 2018). In the technique, replicating DNA is labeled with a thymidine analog (e.g., 5-ethynyl-2’-deoxyuridine, EdU), while DNA fragments containing either new or old histones are immunoprecipitated (via H4K5ac and H4K20me2 marks, respectively). Nascent DNA is subsequently captured via the EdU thymidine analog (see below) and subject to alkaline denaturation to isolate and sequence the newly synthesized strand.

New and old (recycled) histone deposition was also followed in yeast strains expressing Mcm2 mutants that do not bind histones (Gan et al., 2018). Enrichment and sequencing of protein-associated nascent DNA (eSPAN), showed enrichment of old histones on the leading strand as a result of the deficiency. Therefore, Mcm2 is critical for proper histone recycling. In eSPAN, another thymidine analog (5-bromo-2’-deoxyuridine, BrdU) is used to label replicating DNA. This is followed by MNase digestion and ChIP of H3K4me3 (enriched on parental nucleosomes) and of H3K56ac (enriched on new histones in yeast), and followed by strand-specific sequencing (ChIP-ssSeq), as in SCAR-seq. Nascent DNA is mapped back in relation to origins of replication to determine strand identity. Coupled to yeast strains defective for other replication proteins, eSPAN further identified Ctf4 (which links the CMG complex to the lagging strand) and Pol α (which initiates lagging strand synthesis) as additional components that are required for histone recycling on the lagging strand (Gan et al., 2018). Curiously, biophysical analyses indicate that Pol α preferentially binds H2A-H2B (Evrin et al., 2018), highlighting the need for further investigation on mechanistic details of the (H3-H4)_2_ and H2A-H2B recycling at replication forks. Like for the Mcm2 mutant yeast strains, the impairment of this pathway also disrupted gene silencing in yeast (Evrin et al., 2018).

Similarly, recent data implicate the leading strand polymerase ε subunits POLE3-POLE4 in nucleosome assembly. Gel filtration chromatography (which resolves protein complexes based on size) demonstrated H3-H4 binding by human POLE3-POLE4 *in vitro*, while immunoprecipitations confirmed the *in vivo* interaction (Bellelli et al., 2018). Supercoiling assays also demonstrated their ability to promote tetrasome formation *in vitro*, showing *bona fide* histone chaperone activity. This was confirmed *in vivo*, through pulse-chase experiments using fluorescently labeled, SNAP-tagged H3. The SNAP-tag reacts with benzylguanine derivatives and is an efficient way to label and follow proteins. RNAi depletion of POLE3 or POLE4 resulted in a reduced deposition of the tagged histone (Bellelli et al., 2018). Whether additional factors other than MCM2, Pol α, and Pol ε further participate in histone recycling at replication forks remains to be seen.

## Minimal Histone Shuffling at the Fork

Data suggest that recycled histones remain coupled to the replication machinery, and a recently developed *in vitro* system nicely demonstrates this. Biotinylated histones were assembled on a nucleosome positioning sequence at a specific location on a circular DNA template. Their position was then mapped before and after DNA replication (Madamba et al., 2017). Nucleosome positioning was relatively well-preserved when the reaction was driven by eukaryotic (*Xenopus* egg extracts) but not viral (SV40 T-antigen) replication machineries. Techniques have also been developed to track the accuracy of histone redeposition in replicating cells.

Chromatin occupancy after DNA replication by next-generation sequencing [ChOR-seq (Reveron-Gomez et al., 2018)] maps histone distribution on replicated DNA. Akin to iPOND/NCC (described below), cells are briefly pulsed with a recoverable thymidine analog (*e.g.*, EdU) that is incorporated into replicating DNA. ChIP is then performed to isolate chromatin fragments with a specific histone PTM. This allows for the recognition of histone PTMs that are enriched on old, pre-existing histones at either repressed or transcribed regions of the genome. Replicated DNA fragments are further selected and sequenced. ChOR-seq data suggest that old, recycled histones re-incorporate on newly synthesized DNA with surprising fidelity—within some 250 bp of their pre-replication position. Such a tight coupling between recycled histones and the replication machinery is in line with early EM micrographs and biochemical analyses of replicating minichromosomes, showing nucleosomes reassembling some 225–285 bp behind the fork (Herman et al., 1981; Sogo et al., 1986), though the latter could not differentiate between new and recycled histones.

To further follow specific histones through cell division, a biotinylation system akin to that of the *Xenopus* egg extract system (above) was also established in an ESC model (Escobar et al., 2019). Endogenous replication-coupled histone variants were fused to a biotin acceptor peptide (BAP) after which dCas9-BirA was transiently recruited to a specific genomic locus. BirA is a bacterial biotin ligase that recognizes and biotinylates the BAP. A controlled, local biotinylation of histones was therefore achieved. Expression of the dCas9-BirA fusion was tightly regulated and restricted to late G_1_. The biotinylated histones were then followed by ChIP-seq following DNA replication. As per the other studies, the recycled histones remained near their original position after DNA replication. Interestingly, while this was especially true for repressed chromatin regions, histones found on transcribed regions were more apt to disperse in this system. A similar system was also established in yeast, but with BirA fused to the tetracycline repressor TetR (Schlissel and Rine, 2019). Local histone biotinylation was thereby driven by BirA recruitment to an intergenic, single-copy tetracycline operator. ChIP-seq again demonstrated faithful nucleosome redeposition following DNA replication. The biotin ChIP-seq peak remained after rounds of replication, only diminishing in intensity because of the dilution of old biotinylated histones with new non-biotinylated ones. This positional memory was disrupted by the mutation of Mcm2 or depletion of a Pol ε subunit, further highlighting the importance of histone chaperone pathways.

## Post-Replicative Re-Establishment of Histone Marks and Chromatin Maturation

Local chromatin structures need to be re-established following the co-deposition of new and recycled histones on new DNA strands (Figure 4). This maturation process requires, in part, the spreading of at least certain repressive histone marks (*e.g.*, H3K9me2/3 and H3K27me2/3), as well as transcriptional restart (Reinberg and Vales, 2018; Stewart-Morgan et al., 2019).

To further unravel the molecular details by which post-replicative chromatin matures, a number of exciting new proteomic techniques were used. The isolation of proteins on nascent DNA (iPOND) allows researchers to probe the proteome of replicating DNA and post-replicative maturing chromatin (Sirbu et al., 2012). In iPOND, cells are pulsed with EdU, which is incorporated into replicating DNA. When followed by a thymidine chase, it is possible to distinguish newly replicated DNA from maturing chromatin. Proteins that associate with replicating or maturing DNA are then isolated from sheared DNA fragments through the EdU label. EdU contains an alkyne group that is covalently linked to azide coupled moiety (i.e., biotin) *in vitro*, via a copper-catalyzed cycloaddition reaction [“click chemistry” (Gierlich et al., 2006)]. Associated proteins are finally analyzed by western blotting or MS. Different versions of the technique exist; nascent chromatin capture (NCC) coupled to SILAC uses a similar protocol, with a direct comparison of proteins that are associated on nascent versus maturing chromatin (Alabert et al., 2014). As expected, proteins, such as DNA polymerases and the CAF-1 histone chaperone, enriched on nascent chromatin. This elegant technique also led to some intriguing observations. For example, various histone “writers” differently enriched on nascent and mature chromatin. This offers mechanistic insights on the post-replicative re-establishment of histone PTMs.

The analysis of histone PTMs using the NCC-SILAC pipeline showed that there are different propagation modes for different histone PTMs (Alabert et al., 2015). As progression through S-phase caused a twofold dilution of marks, some marks were quickly re-established through G_2_ (e.g., H3K4me3), whereas others took the remainder of the cell cycle or longer (e.g., H3K9me3 and H3K27me3). A progressive, coordinated restoration of histone PTMs was also seen when comparing bulk histone PTM levels by SILAC-MS as synchronized cells progressed through the cell cycle (Zee et al., 2012). The analysis showed that methyl states are not equally reestablished. For example, H3K9me2 resulted from H3K9me1 acquisition of a second methyl group in late-G_1_/S as well as from the acquisition of 2 methyl groups on newly synthesized H3 in G_2_/M. H3K9me3 was in turn established through the addition of a third methyl group to pre-existing H3K9me2 in G_1_/S, and from newly synthesized H3 acquiring 3 methyl groups in G_2_/M. In contrast, H3K27me2 was largely the result of unmodified residues acquiring 2 methyl groups in G_2_/M, while H3K27me3 re-establishment patterns were similar to H3K9me3, but clearly antagonized by the H3K36me3 mark (Zee et al., 2012; Alabert et al., 2020).

In contrast to repressive H3K9me3 and H3K27me3 marks that rely on spreading (Lachner et al., 2001; Margueron et al., 2009), the restoration of the transcription-associated H3K4me3 PTM occurred shortly after DNA replication, with a faster restoration over genomic regions with high transcriptional levels (Reveron-Gomez et al., 2018). Transcription-dependent reestablishment of post-replicative chromatin was further supported by repli-ATAC-seq (Stewart-Morgan et al., 2019), where EdU-labeled replicating DNA is subject to tagmentation, but click chemistry is used to isolate newly replicated fragments. Importantly, the technique showed that, in mESCs, post-replicative chromatin is largely inaccessible until transcription resumes.

## Mitotic Bookmarking: a Brief Primer

Following DNA replication and chromatin maturation, chromatin is condensed in preparation for mitotic segregation of sister chromatids. These changes are accompanied by distinct chromatin alterations that are still under investigation. Mitotic entry is notably characterized by high levels of phosphorylated histones H1 and H3 (Lake et al., 1972; Gurley et al., 1974). The latter is, however, believed to be particularly critical for mitosis (Ohsumi et al., 1993). H3 is specifically phosphorylated at serine 10 (H3S10ph) (Paulson and Taylor, 1982), by the Aurora B (Goto et al., 2002) and VRK1 kinases in mammals (Kang et al., 2007). Phosphorylation begins at the centromere in G_2_, spreads throughout the genome during G_2_/M, and is mostly lost as cells enter telophase (Hendzel et al., 1997). H3 is also phosphorylated at S28 by Aurora B (Goto et al., 2002) beginning in prophase and until anaphase (Goto et al., 1999). The exact roles of these modifications require further study, but they do influence PPIs, and likely assist in a number of mitotic events. For example, HP1 occupancy on chromatin diminishes during mitosis and data indicate H3S10ph prevents HP1 binding (Fischle et al., 2005; Hirota et al., 2005). The condensin I and II complexes, which have been implicated in proper chromatin compaction and mitotic progression, are also thought to be recruited by histone H3 PTMs (Hirano and Mitchison, 1994; Giet and Glover, 2001; Takemoto et al., 2007).

Although it lacks sequence similarity to H3 at its N-terminus, the centromeric H3 variant CENP-A, shares a small stretch of sequence similarity to the H3S10 region and is also phosphorylated after the onset of H3S10ph and through anaphase (Zeitlin et al., 2001). CENP-A is important for mitotic division, and is deposited by the histone chaperone HJURP in late telophase/early G1 (Jansen et al., 2007; Dunleavy et al., 2009). The exact roles of its phosphorylation remain elusive, however, unphosphorylated CENP-A was shown to cause improper microtubule attachment at the kinetochore (Kunitoku et al., 2003). Readers are referred to recent CENP-A reviews for further information on this histone variant (Catania and Allshire, 2014; Muller and Almouzni, 2017).

Data suggest that mitotic “bookmarking” via histone PTMs and chromatin-bound proteins maintains epigenetic information on chromatin as it undergoes profound changes (Kouskouti and Talianidis, 2005; Valls et al., 2005; Young et al., 2007). For example, ChIP-seq in mitotically arrested mESCs demonstrated that H3K27ac was retained at housekeeping gene promoters and stem-cell associated enhancers during mitosis, suggesting that the mark primes transcriptional activation in G_0_/G_1_. Recent data demonstrated H3K4me3 remained associated with most promoters, while H3K27ac was maintained at only a subset of enhancers and promoters, but quickly reestablished at the anaphase/telophase transition (Kang et al., 2020). This was determined using a combinatorial approach with ChIP-seq and EU-RNA-seq, a technique where cells are treated with ethynyl uridine to label newly synthesized RNA, which can then be isolated and sequenced to generate a transcriptome of newly synthesized transcripts. A number of transcription factors, including stem cell regulators, such as SOX2 and OCT4, also remain bound to chromatin in mitosis, which likely contributes to the restoration of transcriptional programs upon mitotic exit (Chen D. et al., 2002; Deluz et al., 2016; Liu et al., 2017). Readers are referred to the following reviews for more information on mitotic bookmarking (Festuccia et al., 2017; Palozola et al., 2019).

## Histone Dynamics During Gene Transcription

**FIGURE 6:**
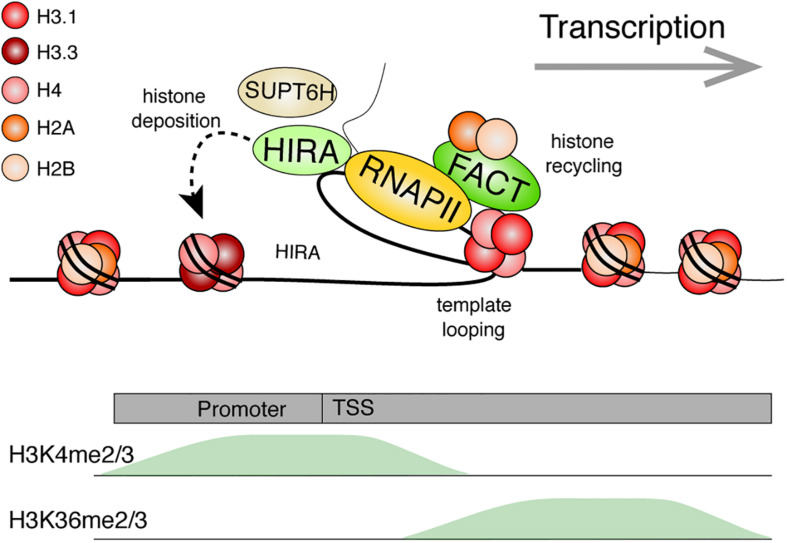
Model: Histone dynamics during transcription. The RNA Polymerase II machinery maneuvers through nucleosomal DNA as it transcribes genes. Histone PTMs are believed to facilitate numerous processes (*e.g.*, splicing or preventing cryptic transcription). Transcribed genes are typically enriched for H3K4me2/3 near the transcriptional start site (TSS) and with H3K36me2/3 within the gene body. The histone chaperone, FACT, facilitates transcription by disrupting histone-DNA contacts and helps preserve nucleosomes by tethering the H2A-H2B dimer while stabilizing the (H3-H4)_2_ tetramer. Template looping is hypothesized to aid in transfer of the tetramer behind the transcriptional machinery. FACT and other histone chaperones including HIRA and SUPT6H further ensure that proper nucleosome density is maintained behind the polymerase through deposition of new histones and aiding in nucleosome reassembly, respectively. References are provided in the main text.

Early studies showed that nucleosomes hinder transcriptional initiation when using *in vitro* transcription systems (Knezetic and Luse, 1986; Lorch et al., 1987). The influence of histones, and their PTMs, toward gene transcription *in vivo* is now amply evident. Experimental models allowing transient histone depletion or the loss of the PTM-rich N-terminal histone tails affect gene expression (Han and Grunstein, 1988; Mann and Grunstein, 1992; Lenfant et al., 1996; Bintu et al., 2012). Over the years, a large number of histone PTMs have been correlated, or anti-correlated, with gene transcription (Barski et al., 2007; Wang et al., 2009). As discussed, H3K4me3 is particularly enriched over the promoter and transcriptional start site (TSS) of active genes, whereas transcribed gene bodies are enriched for H3K36me3 (Bernstein et al., 2005; Talbert and Henikoff, 2006; Wang et al., 2008, 2009; Figure 6).

Chromatin immunoprecipitation nicely captures a nucleosome-free region near the TSS of transcribed genes, and nucleosome phasing at flanking positions (Yuan et al., 2005; Schones et al., 2008). At the promoter, nucleosomes hinder protein binding and chromatin remodeling is often required for efficient transcriptional initiation (Klemm et al., 2019; Brahma and Henikoff, 2020). Once initiated, the elongating transcriptional machinery must then navigate through chromatinized DNA (Kujirai and Kurumizaka, 2020). Nucleosomal histones are disrupted in the process, and mechanisms exist to facilitate transcription while preserving the chromatin environment.

An early *in vitro* study, in which a nucleosome was assembled onto a plasmid, showed that the histones were displaced by the viral SP6 RNA polymerase to reassemble at a different location on the plasmid (Clark and Felsenfeld, 1992). It was suggested that such nucleosome displacement likely involved a direct transfer mechanism without complete histone dissociation from DNA, since competitor DNA had little quenching effect on histones under specific conditions (Studitsky et al., 1994). In addition to histone exchange in the absence of transcription, ChIP, fluorescence recovery after photobleaching (FRAP), and MNase-seq (which, like ATAC-seq, assesses chromatin accessibility) experiments show that H2A-H2B dimer loss and exchange—and to a lesser degree (H3-H4)_2_ exchanges—correlate with active transcription (Kimura and Cook, 2001; Jamai et al., 2007; Cole et al., 2014).

Recent cryo-EM studies revealed that RNA Polymerase II pauses at specific sites within the nucleosome while preserving the histone octamer (Kujirai et al., 2018). Single-molecule force spectroscopy techniques (Gosse et al., 2019), such as optical and magnetic tweezers, enabled controlled biophysical analyses on polymerases and nucleosomes. Using a dual-trap optical tweezer experiment to follow single RNA polymerases on a DNA template containing a single nucleosome, RNA Polymerase II was shown to pause, especially before reaching the dyad axis on the nucleosome (Hodges et al., 2009). DNA looping then facilitated the transfer of histones behind the polymerase.

However, high gene activity appears to be particularly disruptive for nucleosomes (Kulaeva et al., 2010). An *in vitro* system demonstrated this by stalling one or two elongating *Escherichia coli* RNA polymerases ahead of a nucleosome. Stalling was achieved by depleting UTP or UTP and CTP on DNA templates with C and U tracks upstream of the nucleosome. Addition of all nucleotides then allowed transcriptional elongation to occur. The passage of the first RNA polymerase tended to evict an H2A-H2B dimer from some nucleosomes, leaving behind a histone hexamer; whereas the second polymerase could displace the remaining histones (Kulaeva et al., 2010). Nucleosomal density and histone PTMs are, however, maintained over the transcribed gene to facilitate various co-occurring events, and prevent aberrant transcription (Smolle and Workman, 2013). Studies in *Drosophila* indicated that the H3.3 RI histone variant enriches over actively transcribed genes (Ahmad and Henikoff, 2002; Schwartz and Ahmad, 2005), an observation that was then extended to mammals (Ray-Gallet et al., 2011). As mentioned above, nucleosomal density is indeed maintained through transcription-coupled H3.3 deposition, via the HIRA histone chaperone (Goldberg et al., 2010; Sarai et al., 2013).

Additional histone chaperones facilitate transcription while promoting histone recycling at transcribed genes. The histone chaperone FACT is one of the better studied components involved in the disassembly and re-assembly of nucleosomes during transcription. The FACT heterodimer, composed of the SUPT16H and SSRP1 subunits in humans, was first purified based on its ability to facilitate transcription of chromatinized DNA templates *in vitro* (Orphanides et al., 1998). It is, however, important to emphasize that the histone chaperone has since been implicated in numerous other biological events (Gurova et al., 2018). Although first described as an H2A-H2B histone chaperone, it can also bind histones H3 and H4 (Martin et al., 2018; Wang et al., 2018; Mayanagi et al., 2019). FACT allows a controlled assembly and disassembly of nucleosomes *in vitro*. Specifically, recent AUC, optical tweezer, and structural data demonstrate that SUPT16H binds nucleosomal DNA, stabilizes the central (H3-H4)_2_ tetramer, and tethers an H2A-H2B dimer at its DNA binding surface. Meanwhile, SSRP1 facilitates the redeposition of the tethered H2A-H2B dimer, while further maintaining (H3-H4)_2_ on DNA (Chen P. et al., 2018; Wang et al., 2018; Liu et al., 2020). The two subunits synergize to allow for proper disassembly, stabilization, preservation, and reassembly of the nucleosome. Using magnetic tweezers to immobilize and stretch a DNA template containing a single nucleosome, FACT was beautifully shown to promote an orderly histone eviction and reassembly on DNA (Chen P. et al., 2018). Nucleosomes disassembled at lower forces in the presence of FACT. Moreover, the nucleosome did not properly reassemble upon repeated DNA stretching experiments unless FACT was present in the reaction.

Curiously, FACT is not equally expressed across tissues (Garcia et al., 2011), and its loss results in the misregulation of only a subset of transcribed genes (Li et al., 2007). This may, perhaps, be explained by functional redundancy with other histone chaperones. By fractionating nuclear extracts based on the presence of FACT-like activities using an *in vitro* transcription system on chromatinized templates, a recent study identified LEDGF and HDGF2 as novel transcription-coupled histone chaperones (LeRoy et al., 2019). Behind the polymerase, the yeast Spt6 H3-H4 chaperone (SUPT6H in humans), also assists with nucleosome reassembly at highly transcribed genes (Bortvin and Winston, 1996; Ivanovska et al., 2011). ChIP-chip studies in yeast demonstrated that loss of FACT or Spt6 resulted in a transcription-dependent shuffling of evicted histones (Jeronimo et al., 2019). Using the same local histone biotinylation system as with DNA replication, gene induction also led to a gradual loss of histones over the transcribed gene (Schlissel and Rine, 2019). While the ChIP-seq biotin peak diminished in intensity during transcription, its position was largely maintained. Together, this further highlights the important role of histone chaperones in chromatin maintenance.

## Locus-Specific Chromatin Analysis

Rapid advancements in proteomic screening tools now allow us to identify proteins that are bound at specific locations in the genome (Figure 3). ChIP-based experiments have proven instrumental to our understanding of the epigenome, and have also evolved to study PPIs on chromatin. ChIP coupled to western blotting or even MS (ChIP-MS) can identify proteins that are enriched on a chromatin fragment containing an epitope of interest (e.g., a histone mark) (Ji et al., 2015). A more stringent variation of the approach, ChIP-SICAP (Rafiee et al., 2016) identifies chromatin-bound proteins through an initial ChIP, followed by DNA biotinylation and washing, which releases proteins that are not directly bound to chromatin. Though readily applicable, ChIP-based approaches are limited by the quality of the antibodies that are used, and require sufficient material when adapted toward proteomic analyses. The aforementioned ChromID technique (Villasenor et al., 2020) addresses these concerns but will require the design of additional histone-binding modules in order to be tailored for a broader range of experiments investigating protein-protein associations with diverse histone marks.

Equally exciting techniques now allow for locus-specific analysis of chromatin dynamics. To probe sequence-specific chromatin-associated proteins, proteomics of isolated chromatin segments (PICh) was developed (Déjardin and Kingston, 2009). Using locked nucleic acids to hybridize to and isolate sequence specific chromatin fragments and subsequent MS analysis, the authors compared telomeric chromatin composition in cells that use different telomere maintenance mechanisms. The technique has since been adapted to study rDNA (Ide and Dejardin, 2015). While this tour de force proved insightful, it requires a large amount of input material. Protein abundance and the signal-to-noise ratio may also confound results (Gauchier et al., 2020).

Proximity-dependent labeling techniques are yet another promising tool that does not require large amounts of input material, as tags amplify the signal of associated proteins. When fused to a protein of interest (bait), an engineered ascorbate peroxidase (APEX) enzyme (Martell et al., 2012), or promiscuous biotin ligase (e.g., BirA^∗^; a mutant form of the BirA enzyme), labels proximal proteins (preys). Biotinylated proteins are then captured on streptavidin beads and identified by mass spectrometry—a technique known as proximity-dependent biotin identification, or BioID (Roux et al., 2012).

The revolutionary take on prior tagging methodologies (Chen and Ting, 2005) proved immensely powerful. Long labeling times allow the tagging of thousands of interactors per cell compared to AP-MS, which can only capture a snapshot of interactors at a specific time. Therefore, these techniques allow for the amplification and identification of less abundant interactions that traditional pulldowns may miss using comparable input material. While APEX offers rapid labeling, it requires the addition of hydrogen peroxide, which is toxic to cells at high concentrations (Rhee et al., 2013). Biotin ligases such as BirA^∗^ also provide greater signal compared to APEX-based techniques because they target abundant lysine residues, while APEX biotinylates electron-rich residues of lower abundance (e.g., tyrosine). Nevertheless, BioID, and clever derivatives using APEX or biotin ligases, are increasingly utilized to capture stable and biochemically labile interactions, including that of histone proteins (Lambert et al., 2015; Zasadzinska et al., 2018). Interestingly, there is an ongoing effort to use BioID to map the cell where 192 baits from 32 cellular compartments identified over 35,000 unique proximal associations (Go et al., 2019). While insightful, it is also important to recognize that the fusion of two proteins can skew results. Prey proteins also represent proximal and not necessarily direct physical interactions, and BioID data analysis is further influenced by the negative controls to which the data are compared.

Nevertheless, ingenious BioID variations (such as ChromID), continue to be designed [readers are referred to other recent reviews on proximity-based labeling (Kim and Roux, 2016; Samavarchi-Tehrani et al., 2020)]. Of particular interest are those that fuse APEX (CASPEX, C-BERST, and CAPLOCUS) or biotin ligases (CasID) to dCas9, to label proteins at a specific genomic locus, via sgRNA targeting (Schmidtmann et al., 2016; Gao et al., 2018; Myers et al., 2018; Qiu et al., 2019). In initial experiments, CASPEX was localized to the hTERT promoter and identified known interactors, such as TP53 and MAZ (Myers et al., 2018). CasID also proved effective and successfully identified telomeric-bound proteins, such as components of the shelterin complex (Schmidtmann et al., 2016).

## Concluding Remarks

Chromatin is a dynamic structure; nucleosomes can lead to chromatin compaction and occlude accessibility, but also facilitate events, such as gene transcription. In addition to their dynamic nature in G_1_ and G_2_, H3.1, H3.3, and other nucleosomal histones are disassembled, reassembled, or altogether replaced during transcription and DNA replication. However, mechanisms exist to maintain epigenetic features, such as histone PTMs, at precise locations. The intricacies of these processes are still under investigation, but our understanding of them has only been made possible by ever-evolving technologies and the ingenuity of the researchers that develop them.

Here, we highlighted H3 and its processing, PTMs, and interactions. However, other histones, histone variants, and their PTMs also have important biological consequences and these techniques can easily be applied to better understand other histone proteins. The experiments that are showcased are meant to provide a brief overview of the techniques used to study histones and their interactions, and covers but a snippet of available tools. As the chromatin biology field evolves, so will the technology, each time furthering our understanding of histone deposition, modification, binding, and eviction. The growing arsenal of techniques will allow us to continue to dissect histone dynamics through development, transcription, DNA replication & repair, mitosis, and countless other biological events in health and disease.

## Author Contributions

WS wrote the manuscript under the supervision and guidance of EC. All authors contributed to the article and approved the submitted version.

## Conflict of Interest

The authors declare that the research was conducted in the absence of any commercial or financial relationships that could be construed as a potential conflict of interest.
